# Understanding global changes of the mouse brain proteome after vaginal infection with HSV-2 using a label-free shotgun approach

**DOI:** 10.3389/fcimb.2022.942334

**Published:** 2022-08-18

**Authors:** Jishuai Cheng, Qingzhen Wang, Yiwen Hu, Tangwei Mou, Jianbin Wang, Lichun Wang, Ying Zhang, Tinghua Wang, Qihan Li

**Affiliations:** ^1^ Animal Zoology Department, Institute of Neuroscience, Kunming Medical University, Kunming, China; ^2^ Yunnan Key Laboratory of Vaccine Research and Development for Severe Infectious Diseases, Institute of Medical Biology, Chinese Academy of Medicine Sciences and Peking Union Medical College, Kunming, China

**Keywords:** Herpes simplex virus 2, brain, LFQ proteomics, synapse, neurologic disease

## Abstract

Herpes simplex virus type 2 (HSV-2) is a common human pathogen that establishes lifelong latency in neurons of the nervous system. The number of severe central nervous system infections caused by the virus has increased recently. However, the pathogenesis of HSV-2 infection in the nervous system is not fully understood. Here, we demonstrated global proteomic changes in the brain tissue in BALB/c mice vaginally infected with HSV-2. Data are available *via* ProteomeXchange with identifier PXD034186. A total of 249 differentially expressed proteins were identified in infected brain tissue. The GO and KEGG enrichment analysis of these proteins indicated that they were mainly involved in the regulation of synapse formation and synaptic excitability. In addition, genes affecting autophagy, the development of other neurodegenerative diseases, and signaling pathways relevant to other neurologic diseases were identified. Additional experiments, comparing the brain tissue of asymptomatic and symptomatic mice showed a differential expression of proteins involved in synapse formation and synaptic transmission. Others were involved in autophagy, addiction, and signaling pathways of other neurologic diseases. These results suggest that changes in synaptic structure and function, as well as autophagy, may be related to the development of neurologic abnormalities that follow HSV-2 infection. We also identified a protein GluN2A encoded by Grin2a was continuously expressed at high levels after infection. We propose that GluN2A may be a key molecule in the pathogenesis of HSV-2-induced neurologic diseases.

## 1 Introduction

Herpes simplex virus type 2 (HSV-2) a member of the Herpesviridae family, is a ubiquitous human pathogen (Kollias et al., 2015). According to epidemiologic studies, its global prevalence exceeds 25% of the World’s population (Li et al., 2020). HSV-2 can be transmitted through direct contact with seropositive individuals. The virus preferentially infects the skin and mucous membranes, and upon initial contact the virus invades epithelial cells and eventually replicates intracellularly at that site. After initial exposure and resolution of symptoms, the virus lurks in the peri-axonal sheaths of the trigeminal, cervical, lumbosacral, or autonomic ganglion sensory nerves. In these places, viral replication is usually controlled by the patient’s immune system and remains dormant. When reactivation does occur, the virus spreads through sensory nerves to the mucocutaneous site, where it replicates and forms clusters of blisters (Lafferty et al., 1987; Fatahzadeh and Schwartz, 2007; Whitley, 2015). In a subset of viral carriers the reactivation of these dormant viruses can lead to severe, even life threatening, infections, such as viral encephalitis, meningitis, myelitis, or severe neonatal encephalitis (Pinninti and Kimberlin, 2013; Steiner and Benninger, 2013; Wang et al., 2022b). HSV (HSV-1 and HSV-2) infections of the central nervous system (CNS) are devastating and require prompt diagnosis and institution of therapy. Heightened awareness of the disease is essential regardless of whether in a newborn or in an adult. However, there is increasing evidence that HSV infection is associated with a variety of neurological diseases, such as Alzheimer’s disease, Multiple sclerosis, and HSV may be one of its risk factors (Mancuso et al., 2019; Duarte et al., 2021). The pathogenesis of these central nervous system infections caused by HSV-2 infection remains unclear. Although new methods, such as 2-D electrophoresis and Label Free Quantitation (LFQ) have been used to study diseases caused by other pathogens, these approaches have not been utilized to investigate the consequences of HSV-2 infections (Campos et al., 2017; Li et al., 2021). This study used LFQ to quantitatively analyze changes in the brain proteome of BALB/c mice after vaginally infected with HSV-2, and compared proteomics data in samples before and after HSV-2 infection. In addition, differences between symptomatic and asymptomatic infections were also analyzed. Our findings help to identify differentially expressed proteins after HSV-2 infection and screen for key molecules in the nervous system of HSV-2 infection. The functional characterization of these molecules may help to explain the underlying mechanisms causing neurologic abnormalities developing after infection with HSV-2. The data provide insight for further understanding of the CNS infected with HSV.

## 2 Materials and methods

### 2.1 Cells and viruses

The African green monkey kidney cell line, Vero, (ATCC, Rockefeller, MD, USA) was cultivated in Dulbecco’s Modified Eagle Medium (DMEM, Corning, Shanghai, China) containing 10% fetal bovine serum (FBS; HyClone, Glendale, AZ, USA). The medium was changed to DMEM containing 2% FBS after viral inoculation. The wild-type HSV-2 strain, HG52, was purchased from the Wuhan Institute of Virology, Chinese Academy of Sciences (Wuhan, China).

### 2.2 Mice and ethics

Four-week-old female BALB/c mice were purchased (Vital River Laboratory Animal Technology Company, Beijing, China) and housed in a specific pathogen-free facility at the Institute of Medical Biology, Chinese Academy of Medicine Science. Animal experiments were designed in line with the principles described in the “Guide for the Care and Use of Laboratory Animals” and “Guidance for Experimental Animal Welfare and Ethical Treatment”. All animals were under the care of veterinarians at the facility. Experimental protocols were reviewed and approved by the Experimental Animal Ethics Committee of the Institute (approval number: DWSP201803018-1, 1March 2018).

### 2.3 Experimental infection of mice

To induce an acute infection, BALB/c mice were inoculated *via* a vaginal instillation of 5×10^4^ plaque-forming units (PFUs) of HSV-2, with sterile phosphate buffered saline (PBS, pH 7.4) acting as control. Each group Mice were weighed daily and the survival rate-to-mortality ratio was evaluated over 15 days. There were fifteen mice in each group, of which 3 mice were euthanized and dissected for Label-free protein quantitative analysis, viral load and organ pathology (Some results were shown in **Supplement Figure 1**).

### 2.4 Label-free protein quantitative analysis

Proteomics workflow for label-free quantitation of proteins from brains after vaginal infection with HSV-2 was shown in **Supplement Figure 3**.

#### 2.4.1 Sample preparation

After weighing the brain tissue samples PBS was added at a ratio of 1:5 and samples were lysed at low temperature by adding an equal volume of 2× SDT lysis buffer (2% SDS, 0.1 M DTT, 0.1 M Tris/HCL, pH7 at a ratio of 1:1). Vortex homogenization was carried out for 60 s, followed by ultrasonication for 3 min. Samples were heated to 100°C for 10 min to reduce disulfide bonds, centrifuged at 12,000g for 10 min, and the supernatants, containing the total protein, were collected. Protein concentration was measured in each sample by analyzing the fluorescence intensity of tryptophan.

Supernatants containing 100 µg of protein were placed into 10 Kd ultrafiltration tubes to replace the buffer with 8 M UA solution (8 M ready to use UA buffer). After buffer exchange, 100 µL of 50 mM IAA was added, (final IAA concentration not less than 20 mM) and incubated at room temperature, protected from light. The alkylation reaction, blocking sulfhydryl groups, was carried out for 30 min. Next, using 10 Kd ultrafiltration tubes, samples were buffer exchanged into 50 mM NH4HCO3 digestion solution, 4 µg of trypsin was added and samples were incubated overnight at 37°C with continuous shaking. The next day the digested peptide fragments were collected by ultrafiltration. TFA was added to stop digestion, and the ultrafiltrate was desalted on Sep-Pak C18 columns. After the desalting, peptide solutions were dried by a centrifugal concentrator, frozen, and stored at -20°C until use.

#### 2.4.2 Mass detection

Mass spectrometry was performed using a Thermo Scientific™ Q Exactive™ LC/MS system. Samples were applied to a C18 capture column (3 µm, 75 µm × 20 mm, 100 Å) and then eluted to the analytical column (50 µm × 150 mm, 2 µm particle size, 100 Å pore size) for separation. The mobile phases were: phase A: 1% DMSO, 99% H2O, 0.1% formic acid, and mobile phase B: 1% DMSO, 80% ACN, 0.1% formic acid. A 100-min analytical gradient was used as follows: 0 min in 3% B, 7 min of 3%-5%B, 65 min of 5%-18% B, 10 min of 18%-33% B, 2 min of 33%-90% B, and 6 min of 90% B. The flow rate of the liquid phase was 300 nL/min. Mass spectrometry has a variety of scanning modes, including positive and negative ion mode, full scan, etc. In this study, the Data Dependent Acquisition (DDA) scan mode was used. During mass spectrometry DDA mode analysis, each scan cycle included a full MS scan (m/z range 350-1800, ion accumulation time 200 ms), followed by 40 MS/MS scans (m/z range is 100-1500, ion accumulation time 50 ms). The conditions for MS/MS acquisition were set so the precursor ion signal was greater than 3e6, and the charge number was +2~+5. The exclusion time for repeated ion collection was set to 35 s.

#### 2.4.3 Data analysis

The mass spectrum data generated by QE was retrieved with Protein Discover (V2.2). The mass spectrometry proteomics data have been deposited to the ProteomeXchange Consortium *via* the PRIDE (Perez-Riverol et al., 2022) partner repository with the dataset identifier PXD034186. The Percolator database retrieval algorithm was used with the mouse proteome reference Uniprot database (UniProt Mouse 20180428. fasta). Search parameters were as follows: Scan Event: Mass Analyzer (FTMS), MS Order (MS2), Activation Type (HCD), Min. Collision Energy (0), Max. Collision Energy (1000), scan type (full); Sequest HT: Enzyme (Trypsin full), Max. Missed Cleavage Sites (2), Min. Peptide Length (6), Max. Peptide Length (144), Dynamic Modification (Oxidation, Acetyl, Carbonadomethyl); Percolator: Max. Delta Cn (0.05), Maximum Rank (0), Target FDR (Strict) (0.01), Target FDR (Relaxed) (0.05) and Validation based on q-Value. Entries and contaminating proteins present in the anti-database were deleted, and the remaining identification information was used for subsequent analysis.

##### 2.4.3.1 Screening for differentially expressed proteins

Mass spectrometry detected the scores, peptides, and spectral numbers of obtained proteins in the samples. Due to the higher protein abundance, a large number of profiles were collected, allowing the subsequent relative quantitative detection of differentially expressed proteins between groups. A sum PEP score ≥ 1.5 protein was selected and contaminating proteins were removed. After database alignment search, the mass number deviation of retrieved peptides followed normal distribution. Based on the number of detected peptide spectra and scores of mass spectrometry, screening Adj. P-Value (PV) <0.01, |Log2 FoldChange (FC)|>1.

##### 2.4.3.2 Differential protein analysis

In the previous step, we screened the proteins according to PSM score ≥ 1.5, and screened the differential proteins of Day3 and Day7 groups compared with Control according to Adj. P-Value (PV) <0.01, |Log2 FoldChange (FC)|>1. To test the plausibility and accuracy of differentially expressed molecules the detected proteins were aggregated together based on the trend of expression levels using cluster analysis. To establish the functional characteristics of differentially expressed proteins, Gene Ontology (GO) analysis (Lyu et al., 2022) and Kyoto Encyclopedia of Genes and Genomes (KEGG) pathway enrichment analysis (Kanehisa and Goto, 2000) were performed using the Metascape database. The GO and KEGG analysis parameter p-value cutoff was set to 0.05 with a minimum number of matches of 3 and a minimum enrichment score of 1.5 Results were presented using the ImageGP platform.

##### 2.4.3.3 Protein-protein-interaction (PPI) network analysis

To clarify the interactions between 249 differentially expressed proteins, we constructed the protein-protein interaction (PPI) network based on an online resource STRING (Ver. 11.5) database (http://string-db.org) (Szklarczyk et al., 2021). The minimum interaction cut-off standard was set to 0.4 (medium interaction confidence score). Cytoscape software (Ver. 3.7.2) was utilized for visualization. At the same time, one of the proteins of interest was annotated based on the results of GO and KEGG.

## 3 Results

### 3.1 HSV-2 infection causes significant changes in brain proteome

**Figure 1:**
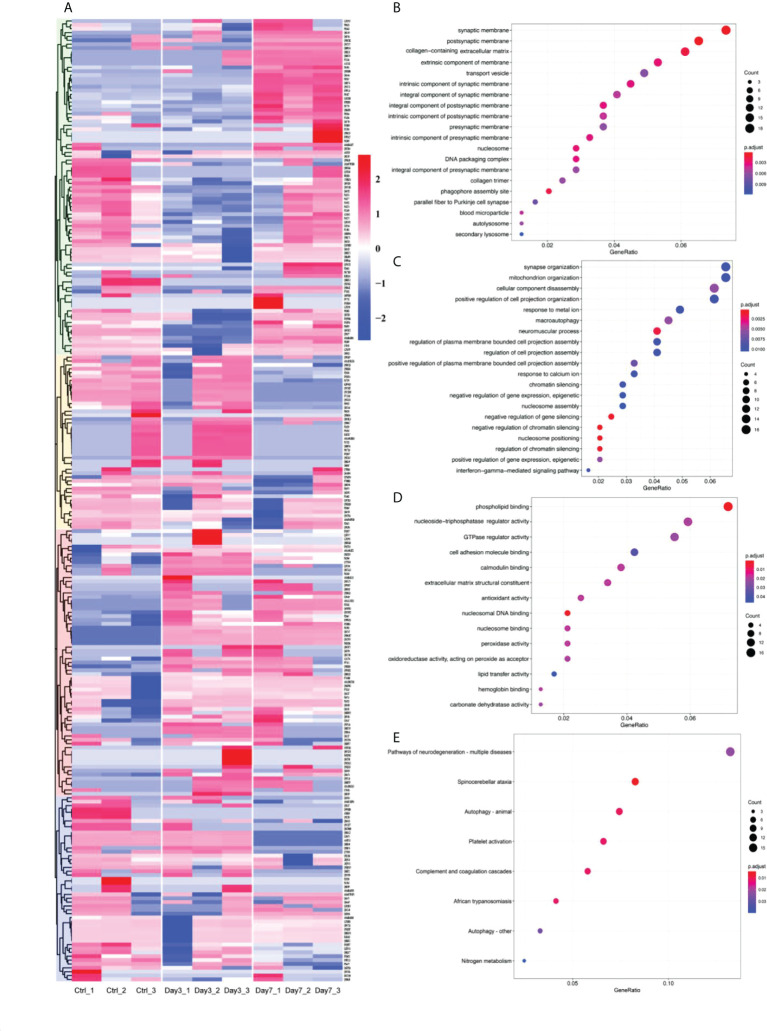
Altered protein expression in brain induced by vaginally infection with HVS-2. **(A)** Heatmap of differentially expressed proteins. Red represents upregulated proteins, blue downregulated proteins, with more intense colors representing more pronounced differences. **(B-D)** Partial results of GO function enrichment analysis, **(B)** affected cellular components, **(C)** biological process, and **(D)** molecular function. The size of circles is proportional to the level of enrichment. Red indicates the highest, blue indicates the lowest P value. **(E)** Some of the results of KEGG pathways enrichment. Again, the size of circles corresponds to the level of enrichment. Red indicates the high, blue low P values.

To investigate pathologic changes in brain tissue after vaginal infection with HSV-2, we analyzed total protein samples with spectroscopy, identifying and quantitating proteins from brain tissues before and after infection. Proteins with sum PEP score ≥ 1.5 were detected, while eliminating contaminating proteins. This analysis identified 3546 proteins. Of these 3242 were present in the brains of control (Ctrl), non-infected animals. 3256 proteins could be identified in samples 3 days after HSV-2 infection, while 3241 proteins were detected at the 7-day timepoint. Differentially expressed proteins (DEPs) were screened out according to the following criteria: adj P-Value<0.01 and |Log2FoldChange|>1. Compared to control brains, there were 249 DEPs detected in HSV-2 infected samples, with some notable differences between protein expression seen on day 3 and day 7 of the infection (See **Supplement Table 1**). These differentially expressed molecules were subjected to an initial cluster analysis (See **Figure 1A**). In order to gain some insight into the function of these proteins we performed Gene Ontology (GO) and Kyoto Encyclopedia of Genes and Genomes (KEGG) analysis. GO enrichment results show that these differential proteins are mainly involved in postsynaptic membrane, synaptic membrane, intrinsic component of synaptic membrane, intrinsic component of presynaptic membrane and other cell components, synapse organization, positive regulation of cell projection organization, response to calcium ion and other biological processes, and molecular functions such as phospholipid binding, calmodulin binding, nucleoside-triphosphatase regulator activity. And the KEGG enrichment results showed that these differential proteins were mainly involved in signaling pathways such as Spinocerebellar ataxia, Complement and coagulation cascades, Pathways of neurodegeneration - multiple diseases, Autophagy. The results of GO and KEGG analysis indicated that both the structure and the function of synapses were affected by the infection. There was also evidence for changes in genes involved in autophagy, gene regulation, and immunological responses (See **Figures 1B–E**). These changes could potentially explain neurologic abnormalities after the infection of the nervous system, indicating that HSV-2 infection might cause neurodegenerative changes.

### 3.2 Significant difference in brain proteome between day 3 and day 7 of HSV-2 infection

**Figure 2:**
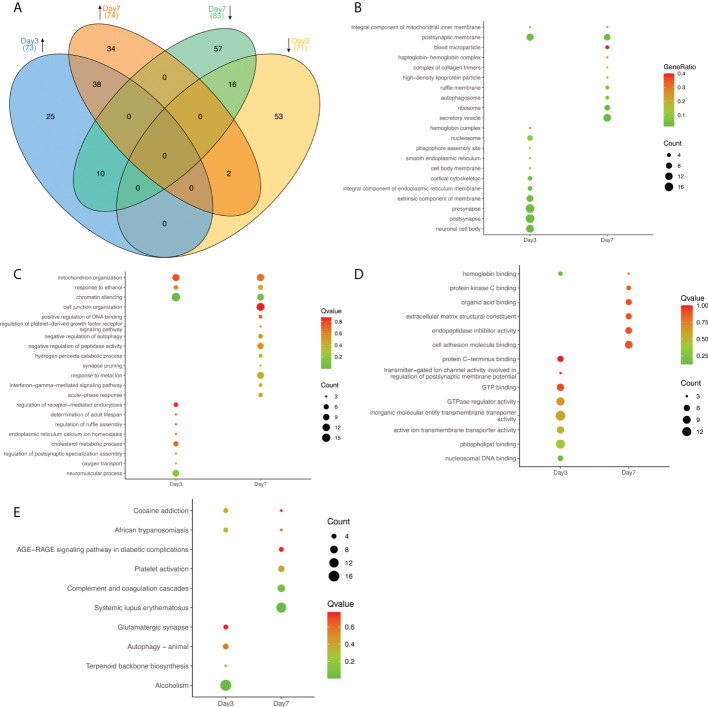
The expression of representative proteins in the infected brains at Day 3 and Day 7. **(A)** Venn diagram illustrating the relationships of proteins at various timepoints. Upward pointing arrows indicate upregulation, whilst downward pointing arrows illustrate downregulation. Numbers in brackets represent the number of proteins in each subset. **(B–D)** Some representative results of GO function enrichment amongst differentially expressed proteins, **(B)** affected cellular components, **(C)** biological process, and **(D)** molecular function. The size of individual circles corresponds to the number of enriched proteins. Red indicates the highest, blue the lowest P value. **(E)** Partial results of KEGG pathways enrichment in DEPs. The size of circles represents the number of proteins in a group. Red indicates the highest Q value, green indicates the lowest Q value.

Cluster analysis of DEPs identified different expression signals at 3 days and 7 days after infection. Mice showed no obvious clinical symptoms on day 3 of the acute infection, while by day 7 obvious clinical neurologic symptoms became evident, such as hindlimb paralysis, became evident. To explore pathologic mechanisms after HSV-2 infection, we identified and analyzed proteins in mouse brain tissues at both time points. Compared to the control brains, 144 proteins showed significant differences in expression in the Day 3 group, while 157 DEPs were detected in the Day 7 group. These data are summarized in **Figure 2**. The Venn diagram indicated there was a varying degree of intersections between each pair of groups (**Figure 2A**). A total of 73 upregulated proteins were see in the Day 3 group. Of these, 25 proteins were only upregulated at 3 days after HSV-2 infection and returned to normal levels by day 7. A further subset of 10 proteins was initially upregulated on day 3, but became downregulated by day 7. It is of note that there were six proteins (Chm, Tgm3, Arhgap24, Tanc1, Agap1, Pnkd) that were uniquely expressed on the third day after infection. Of 71 day-3 downregulated DEPs, 53 proteins returned to normal later, while the expression of an additional two proteins was upregulated by day 7. In the Day 7 group, 34 of 74 proteins upregulated DEPs were selectively augmented their abundance at this time point only. Furthermore, there were nine proteins that were specifically detected at 7 days post-infection (Saa2, Trappc1, Cxadr, Armc8, Hbxip; Lamtor5, Prppsap2, Usp46, Pcdh10, Neo1). In contrast, 83 proteins were downregulated, with the expression of 57 DEPs altered at 7 days (See **Figure 1A** for details).

As summarized in **Figure 2B**, GO analysis showed that DEPs at day 3 mostly contained those contributing to presynaptic (17), postsynaptic (17) and neuronal cell body (13) structures. By day 7 the most prominently affected cellular structures were secretory vesicles (13). Analyzing the involved biological processes, DEPs were enriched in mitochondrial organization, response to ethanol, and chromatin silencing. Genes involved in neuromuscular processes (10), the modulation of chemical synaptic transmission (11), and the regulation of ion transport (13) were seen at 3 days after infection. By day 7 proteins involved in responses to metal ion (11), the regulation of plasma membrane bounded cell projection organization (13), and cell junction organization (14) were the most prevalent (See **Figure 2C**). Regarding molecular function, DEPs were enriched for hemoglobin binding at both time points. On day 3 the main enriched molecular functions were GTPase regulator activity (10), phospholipid binding (12), and inorganic molecular entity/transmembrane transporter activity (14). By 7 days cell adhesion molecule binding (9) and endopeptidase inhibitor activity (7) were the most predominant features (See **Figure 2D**). In the KEGG signaling pathway analysis, proteins were enriched in cocaine addiction and African trypanosomiasis signaling pathways at both time points. The 3-day DEPs were relevant to alcoholism (17) significantly but tipped in favor of systemic lupus erythematosus and the complement and coagulation cascades (8) at the 7-day time point (See **Figure 2E**). The results indicated that HSV-2 infection shared some similarities with certain neurological diseases. Not unexpectedly, the brain infection caused by HSV-2 was associated with genes involved in immune responses and inflammation. We screened 8 proteins involved in complement and coagulation system, which are encoded by the following genes: C1qb, C1qc, C3, Cfh, Fga, Kng1, Fgg, Fgb, Dsg1b. The complement and coagulation cascades are evolutionarily related proteolytic cascades in the blood that are critical for appropriate innate responses to injury limiting bleeding and infection, while promoting healing. Although often seen as distinct, it has long been recognized that there may be crosstalk between these pathways, and molecular connections have only recently been established (Maloney et al., 2020). These details provided insights provide an opportunity to develop new therapeutic strategies to better treat a wide range of thrombotic, inflammatory, immune, infectious and malignant diseases (Conway, 2018).

We identified that the expression of Glutamate receptor ionotropic, NMDA 2A (gene symbol: Grin2a) continued to increase at 3- and 7-days post-infection. Our GO annotation pointed out that this protein is a component of the NMDA receptor complex, as a heterotetrameric, ligand-gated ion channel with high calcium permeability and voltage dependence on magnesium Sexual sensitivity, mainly involved in molecular functions such as ligand-gated ion channel activity, glutamate receptor activity, and ion channel activity regulated by pre- and post-synaptic membrane potential, and biological processes such as synaptic plasticity, synaptic transmission, and ion transmembrane transport, and contributes to excitatory postsynaptic currents, long-term synaptic potentiation, and the slow phase of learning, thought to play a role in neurodegenerative diseases (Meguro et al., 1992; Sakimura et al., 1995). These results suggest that the protein GluN2A encoded by Grin2a may play an important role in HSV-2 infection of brain tissue.

### 3.3 Protein-protein interaction network analysis

**Figure 3:**
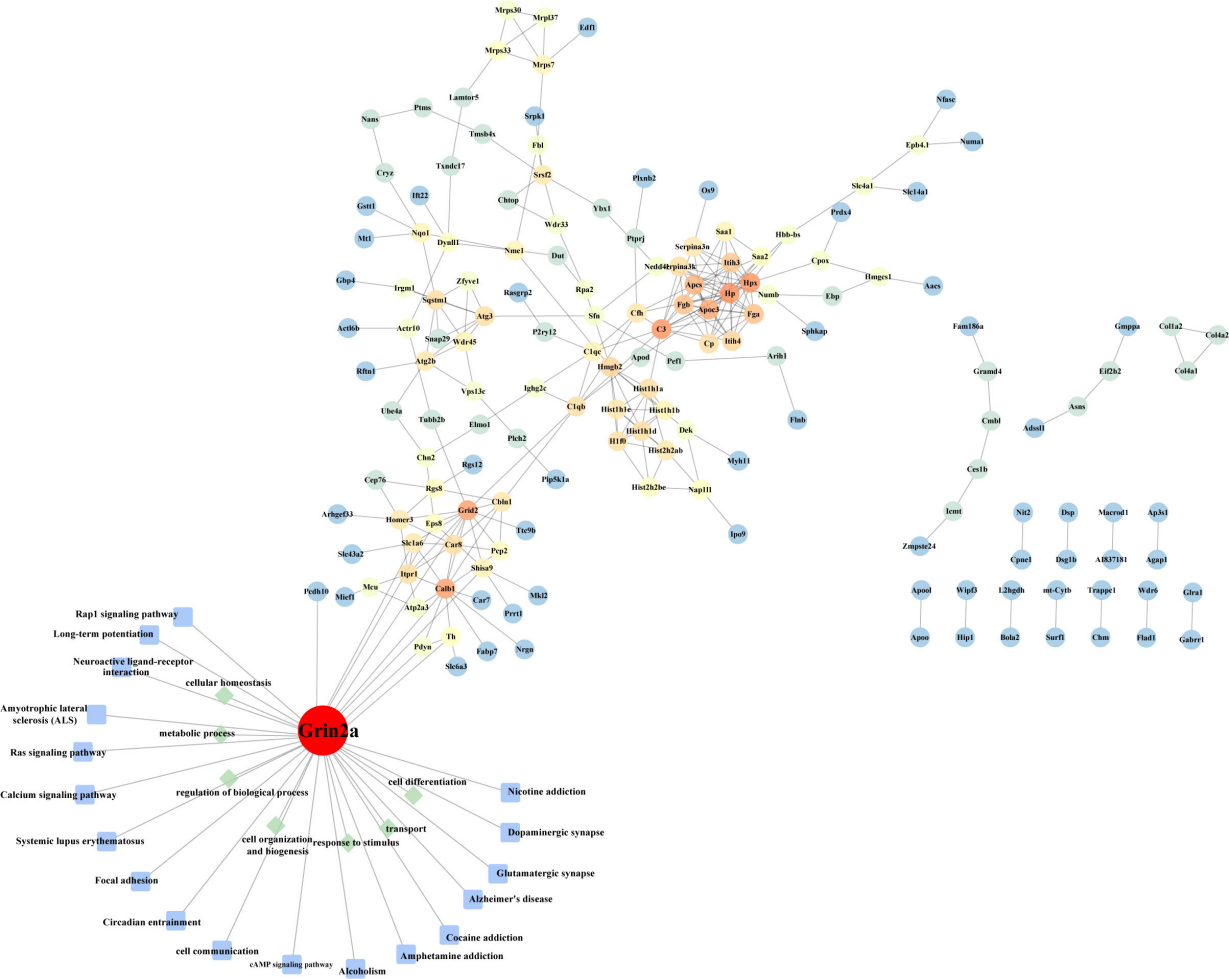
Protein-protein interaction network. The differentially expressed proteins in the heatmap were subjected to build a protein interaction network. GO and KEGG annotations were performed on the molecule GluN2A (encoded by Grin2a) we screened.

The construction of protein-protein interaction networks (PPI network) can help in understanding molecular mechanisms of disease development and may lead to the discovery of potential new drug targets. A network of 161 nodes and 266 edges was constructed based on the DEPs (See **Figure 3**). We observed several targets that had direct interaction with the one of interest, Grin2a, such as Slc1a6, Grid2, and Shisa9, all of which are associated with synapses. Slc1a6 Plays a redundant role in the rapid removal of released glutamate from the synaptic cleft, which is probably essential for terminating the postsynaptic action of glutamate. Grid2 is located in dendritic spine and postsynaptic membrane, is integral component of postsynaptic density membrane and is a part of ionotropic glutamate receptor complex which is active in glutamatergic synapse and parallel fiber to Purkinje cell synapse. Shisa9 is a regulator of short-term neuronal synaptic plasticity in the dentate gyrus. Associates with AMPA receptors (ionotropic glutamate receptors) in synaptic spines and promotes AMPA receptor desensitization at excitatory synapses. These results again suggest that GluN2A may be a key molecule in the pathological changes of brain tissue induced by HSV-2 infection.

## 4 Discussion

HSV-2 is one of the most prevalent sexually transmitted diseases in the world (Feng et al., 2021). In addition to causing recurrent genital herpes, the virus is also latently present in sacral dorsal root ganglia. Reactivation of these latent viral particles can cause serious neurological diseases, including HSV-2 meningitis, neonatal Herpes simplex virus type 2 encephalitis (Hayes et al., 2022). The presented work aimed to determine quantitative changes in the brain proteome of HSV-2-infected mice using proteomics analysis methods. Proteins that play characteristic roles during infection in the brains of infected animals were also analyzed. Large-scale protein profiling enables comparative measurements of multiple proteins simultaneously, while label-free proteomics methods are optimized to detect small changes in protein abundance (Campos et al., 2017). Therefore, we were able to detect small changes in protein abundance in infected and non-infected brain samples with stringent criteria. As previously reported, mice infected with HSV-2 initially showed no clinical symptoms until the viral load increases beyond a certain level. We confirmed that BALB/c mice remained asymptomatic for the first 3 days after vaginal inoculation. Symptoms started to appear at 4 days after infection, and became apparent 7 days later, including hindlimb paralysis and death. The replication of the virus in the brain was also different between the two stages (See **Supplement Figure 1**). In the study of proteomic changes, we selected day 3 to represent the asymptomatic period and day 7 after the infection to represent the symptomatic period.

Importantly, heatmap demonstrated that there were significantly differences in the expression of 249 proteins between non-infected and HSV-2 infected brains, with notable differences of 297 proteins in the brain tissue of asymptomatic and symptomatic animals. Gene ontology categorization revealed that these proteins were involved in various of cellular processes. Synaptic composition was affected, with alteration in the protein components and structural features of both pre- and postsynaptic membranes. These clues support the synapse, a key site where the functional connection between neurons occurs (Faria-Pereira and Morais, 2022), whose information transmission was in chaos post infection. Alterations in synaptic signaling, in the release of neurotransmitters from presynaptic neurons, changes in binding to postsynaptic receptors, and synaptic degeneration are evident in the course of many neurodegenerative diseases (Parmar et al., 2022; Peng et al., 2022; Wang et al., 2022a). Indeed, we found that several differentially expressed proteins were involved in the pathways of KEGG associated with neurodegenerative diseases, autophagy, and other signaling. These results suggest that HSV-2 infection may have some similarities with neurodegenerative diseases.

Notably, there is growing epidemiological and experimental evidence suggest that recurrent HSV infection is a risk factor for Alzheimer’s disease (AD) (Shinomoto et al., 2021; Protto et al., 2022), although the underlying molecular and functional mechanisms have not been fully elucidated. Herpes simplex virus is a neurotropic double-stranded DNA virus that primarily infects the epithelial cells of the oral cavity, nasal mucosa, and vagina. Here, the virus undergoes replicates, and newly generated viral particles may enter sensory neurons and transport to the trigeminal and dorsal sacral root ganglia where latent infection is established (Yun et al., 2014). The virus undergoes periodic reactivation in which newly formed viral particles are transported back to the primary site of infection *via* sensory neurons, resulting in well-known clinical lesions (ie, herpes and blisters). The virus can reach the central nervous system (CNS) from latently infected ganglion neurons, where it may cause acute neurological disease such as encephalitis or mild, clinically asymptomatic infection, or establish lifelong latent infection (Rozenberg, 2020). The weakened immune system that occurs during aging may favor this process. Burgos et al. demonstrated that in addition to the neuronal pathway, HSV-1 can enter the central nervous system *via* the bloodstream. Several reports suggest that during infection, herpesviruses interact with several human proteins which are used to enter cells and move from the plasma membrane to the nucleus and back. HSV also utilizes the host’s transcriptional machinery to replicate and bind proteins that control immune surveillance or apoptosis. Interestingly, in an attempt to eliminate the virus, the host may even cause cellular damage through immune and inflammatory responses against virus-containing cells. If this occurs in the CNS, HSV-induced inflammatory responses may lead to HSE or, in less severe cases, cell death and neurodegeneration (Piacentini et al., 2014). In a 14-year follow-up of 512 elderly patients without initial dementia, Letenneur et al. showed that serum anti-HSV IgM-positive subjects had a significantly higher risk of AD (hazard ratio 2.55) and serum anti-HSV IgG-positive subjects did not. Significantly increased risk of AD was found (Harris and Harris, 2015). Localization and autopsy studies of patients with HSV-1 encephalitis have found that HSV-1 is specifically neurotropic in the temporal cortex, frontal lobe, and hippocampus, the same regions of brain affected by AD (Steel and Eslick, 2015). *In vitro* and animal studies have shown that HSV-1 infection leads to the development of the main features of AD, especially neuroinflammation, Amyloid β-peptide (Aβ) deposition and Tau protein phosphorylation, and the accumulation of Aβ may be due to its potential antiviral effect (Harris and Harris, 2018). HSV-2 infection results in the accumulation of hyperphosphorylated Tau and β-amyloid in human SK-N-MC neuroblastoma cells. HSV-1 remains dormant after initial infection and periodically reactivates as immunity declines (Bellon and Nicot, 2017). Long-term immune senescence may result in more frequent and/or intense reactivation of the virus with age. The gradual onset and relatively late onset of AD can be explained in terms of time (Stowe et al., 2012). These evidences suggest that the accumulation of intracellular damage caused by HSV viral infection may be associated with neurodegeneration and even Alzheimer’s disease, particularly viral reactivation.

In this case, we captured GluN2A, a vital protein associated with neurological function as well as HSV-2 infection, which also participates the progression of AD. The characteristic pathological changes of AD mainly include the accumulation of Aβ and the formation of extracellular neuroinflammatory plaques and the hyperphosphorylation of Tau (Ittner and Gotz, 2011; Guan et al., 2022) Shrinking, massive loss of neurons in the brain area and abnormal synaptic function, cause progressive cognitive decline (such as memory loss, loss of cognitive ability, etc.) and behavioral impairment in patients (Lane et al., 2018). As an early event in AD pathogenesis, synaptic degeneration is closely related to cognitive impairment in AD patients. Evidence suggests that Aβ is a major factor in synaptic dysfunction and loss in the brains of AD patients. Early studies have shown that Aβ interferes with synaptic function by binding to ionotropic glutamate N-methyl-D-aspartate receptors (NMDARs) as a gain-of-function ligand, mediating physiological synaptic plasticity and glutamate acid-induced neurotoxicity (Chang et al., 2016). NMDARs are encoded by Grin and exist as heterotetrameric complexes consisting of two essential GluN1 and two GluN2 (Guan et al.) and/or GluN3 (A-B) subunits. NMDARs play an important role in neural development and formation, plasticity of learning and memory, and cell survival (Gurma et al., 2021) and their dysfunction is closely related to many neurological diseases, such as Alzheimer’s disease and Parkinson’s disease. The function of NMDARs is determined by the difference of GluN2 subunits (Wyllie et al., 2013). Among these subunits, the GluN2A protein encoded by Grin2a is present in the nerve cells (neurons) of the brain and spinal cord, and is mainly expressed in the cortex and hippocampus, which are closely related to learning/memory, and is distributed on the postsynaptic membrane and is increasing with age gradually (Booker and Wyllie, 2021). Other researcher found that astrocyte GluN2A subunit exerts neuroprotective effects by promoting synaptic survival and identified nerve growth factor as a mediator of astrocyte GluN2A buffering Aβ synaptic toxicity (Li et al., 2016). In this study, during the acute infection phase of the HSV-2 mouse model of vaginal infection, the quantitative analysis of LFQ protein showed that the expression of GluN2A encoded by Grin2a increased in the mouse brain tissue, but no significant changes in the expression of Aβ were detected. Based on this, we speculate that GluN2A may be a key molecule in the neuroprotection of HSV-2 during acute infection, and may also be a key molecule unrelated to Alzheimer’s disease during acute infection.

## 5 Conclusion

In this work, we conducted a quantitative shotgun proteomic analysis of proteins in brain tissues of BALB/c mice in the acute phase after vaginal infection with HSV-2. The results showed that the differentially expressed proteins during the acute infection were mainly involved in the formation of synaptic structure and the regulation of synaptic excitability, and were related to a variety of neurological diseases, such as neurodegenerative diseases. Changes in synaptic structure and function may be the reasons for the occurrence of neurological diseases after infection, and the screened protein GluN2A may play an important neuroprotective role in neurological diseases caused by HSV infection, which remain to be further studied. These data provide further evidence that HSV is a risk factor for Alzheimer’s disease. Of course, given the growing evidence that reactivation of HSV infection is associated with Alzheimer’s disease, this study only studied the acute infection period, so there are certain limitations.

## Data availability statement

The data presented in the study are deposited in the ProteomeXchange Consortium via the PRIDE partner repository,accession number PXD034186.

## Ethics statement

The animal study was reviewed and approved by Animal Ethics Committee, Institute of Medical Biology, Chinese Academy of Medical Sciences.

## Author contributions

QL and TW conceived and designed the study. JC and QW performed the experiments and analyzed the data. YH contributed data curation and manuscript modification. TM, JW, LW, and YZ contributed reagents and materials. JC wrote the first draft. All authors contributed to the article and approved the submitted version.

## Funding

This work was financially supported by the National Natural Science Foundation of China (grant numbers 31670173 and 82171817), the funds for Scientific Research from Department of Education of Yunnan Province (grant number 2022J0205), the funds for 2022 Open Project of Yunnan Provincial Key Laboratory of Clinical Virology (grant number 2022A600301), and the funds for IMBCAMS PhD Innovation (grant number 2018018004).

## Acknowledgments

We would like to thank Wuhan Servicebio Technology Co., Ltd. for technical help.

## Conflict of interest

The authors declare that the research was conducted in the absence of any commercial or financial relationships that could be construed as a potential conflict of interest.

## Publisher’s note

All claims expressed in this article are solely those of the authors and do not necessarily represent those of their affiliated organizations, or those of the publisher, the editors and the reviewers. Any product that may be evaluated in this article, or claim that may be made by its manufacturer, is not guaranteed or endorsed by the publisher.

## Supplementary material

Supplementary Figure 1 Clinical symptoms in mice after vaginally infection with HSV-2. **(A)** Survival rate following vaginal challenge with HSV-2. **(B)** Weight following vaginal challenge with HSV-2. **(C)** Viral loads were determined in the brain from HSV-2 vaginally infected mice. The data are shown as the mean ± SD. Statistical significance was measured by the log-rank test. * p < 0.05, ** p < 0.01, ***p < 0.001. **(D-F)** Pathological changes in the brain of mice infected with HSV-2 *via* vaginal challenge. D shows the brain of mice in Ctrl group, E shows the brain of mice in Day3 group, F shows the brain of mice in Day7 group. Hematoxylin-eosin staining **(D–F)**; Bar = 100 μm. In F, the interstitial structure of neurons in the brain tissue is loose, and a large number of neuron cells are contracted and stained, as shown by the black arrow; necrosis and liquefaction can be seen in a small area, as shown by the red arrow; accompanied by a small amount of hemorrhage, as shown by the yellow arrow.

Supplementary Figure 2 The expression of two proteins differentially expressed in brains after vaginal infection with HSV-2. We selected the protein Amyloid-beta A4 protein (Aβ) encoded by App and the protein Caveolae-associated protein 2 encoded by cavin2 and detected the expression levels of these proteins. The results showed that the changes of the expression levels of these proteins after HSV-2 infection were consistent with the quantitative results of mass spectrometry. The data are shown as the mean ± SD. * p < 0.05.

Supplementary Figure 3 Proteomics workflow for label-free quantitation of proteins from brains after vaginal infection with HSV-2.
