# Supplementary figures and images for: Understanding global changes of the mouse brain proteome after vaginal infection with HSV-2 using a label-free shotgun approach
Source: Front Cell Infect Microbiol. 2022 Aug 18;12:942334. doi: 10.3389/fcimb.2022.942334 (PMC9433710; doi:10.3389/fcimb.2022.942334)

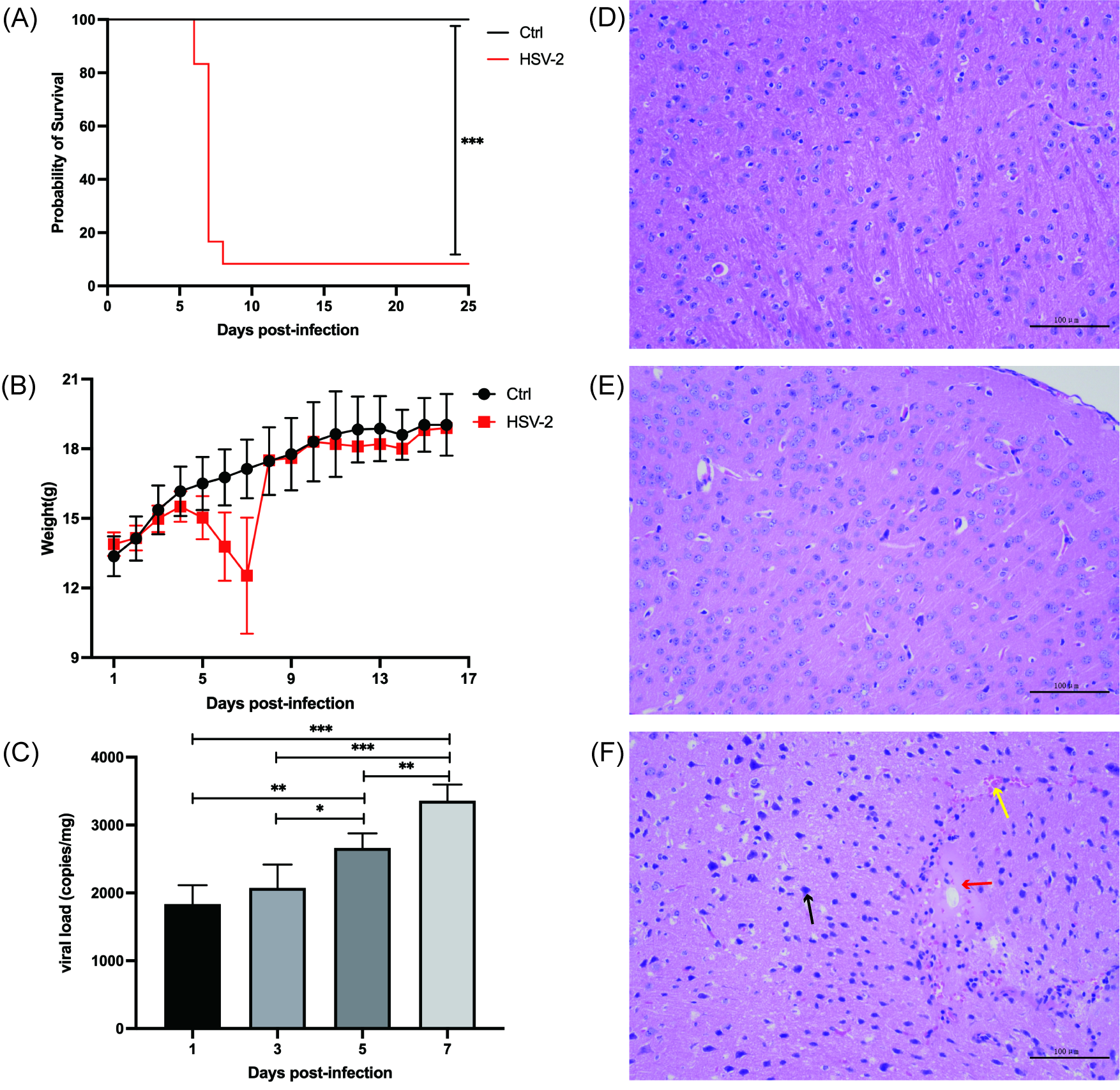

Supplement: Supplementary Figure 1 — Clinical symptoms in mice after vaginally infection with HSV-2. (A) Survival rate following vaginal challenge with HSV-2. (B) Weight following vaginal challenge with HSV-2. (C) Viral loads were determined in the brain from HSV-2 vaginally infected mice. The data are shown as the mean ± SD. Statistical significance was measured by the log-rank test. * p < 0.05, ** p < 0.01, ***p < 0.001. (D-F) Pathological changes in the brain of mice infected with HSV-2 via vaginal challenge. D shows the brain of mice in Ctrl group, E shows the brain of mice in Day3 group, F shows the brain of mice in Day7 group. Hematoxylin-eosin staining (D–F); Bar = 100 μm. In F, the interstitial structure of neurons in the brain tissue is loose, and a large number of neuron cells are contracted and stained, as shown by the black arrow; necrosis and liquefaction can be seen in a small area, as shown by the red arrow; accompanied by a small amount of hemorrhage, as shown by the yellow arrow. [file Image_1.tif]

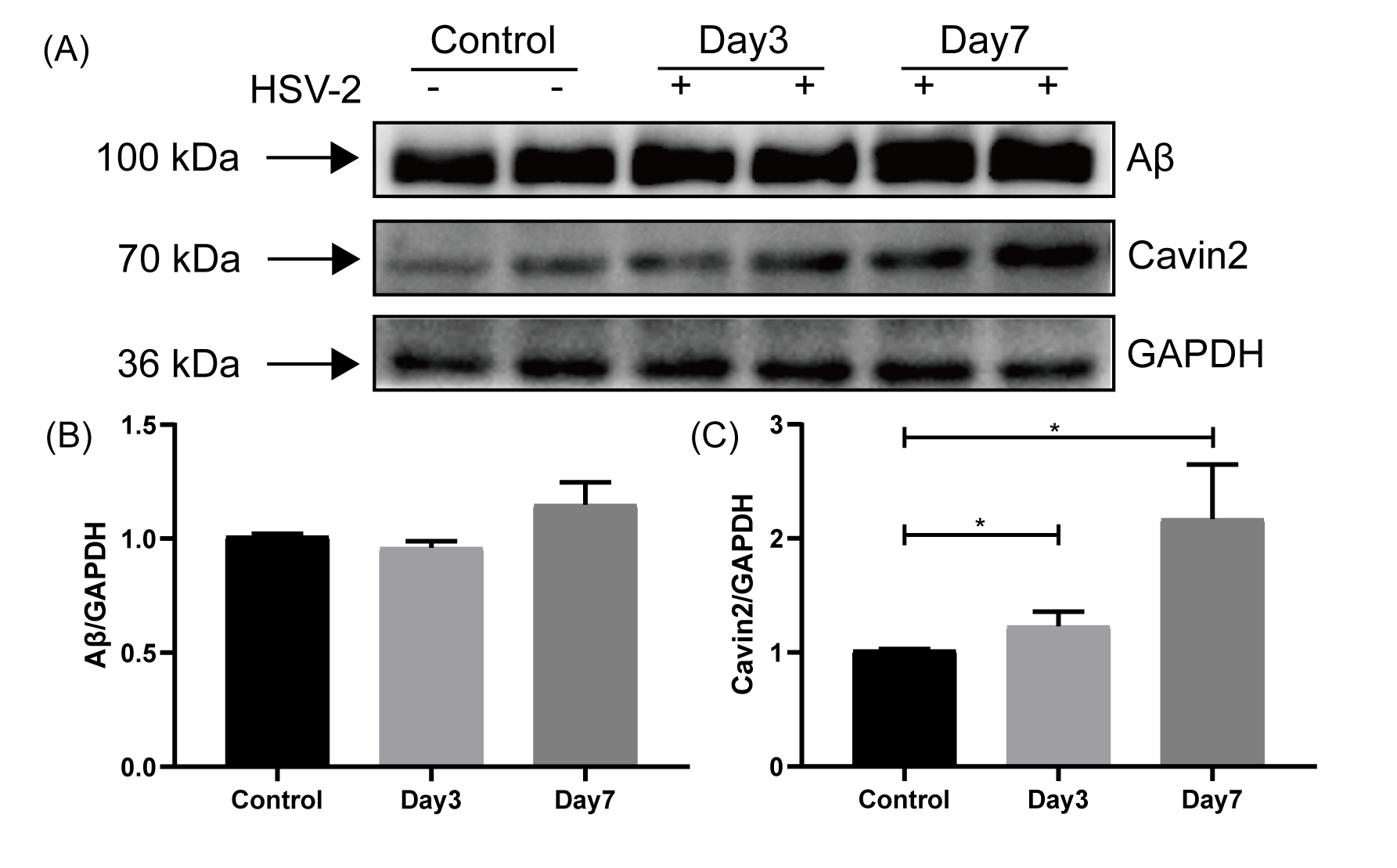

Supplement: Supplementary Figure 2 — The expression of two proteins differentially expressed in brains after vaginal infection with HSV-2. We selected the protein Amyloid-beta A4 protein (Aβ) encoded by App and the protein Caveolae-associated protein 2 encoded by cavin2 and detected the expression levels of these proteins. The results showed that the changes of the expression levels of these proteins after HSV-2 infection were consistent with the quantitative results of mass spectrometry. The data are shown as the mean ± SD. * p < 0.05. [file Image_2.tif]

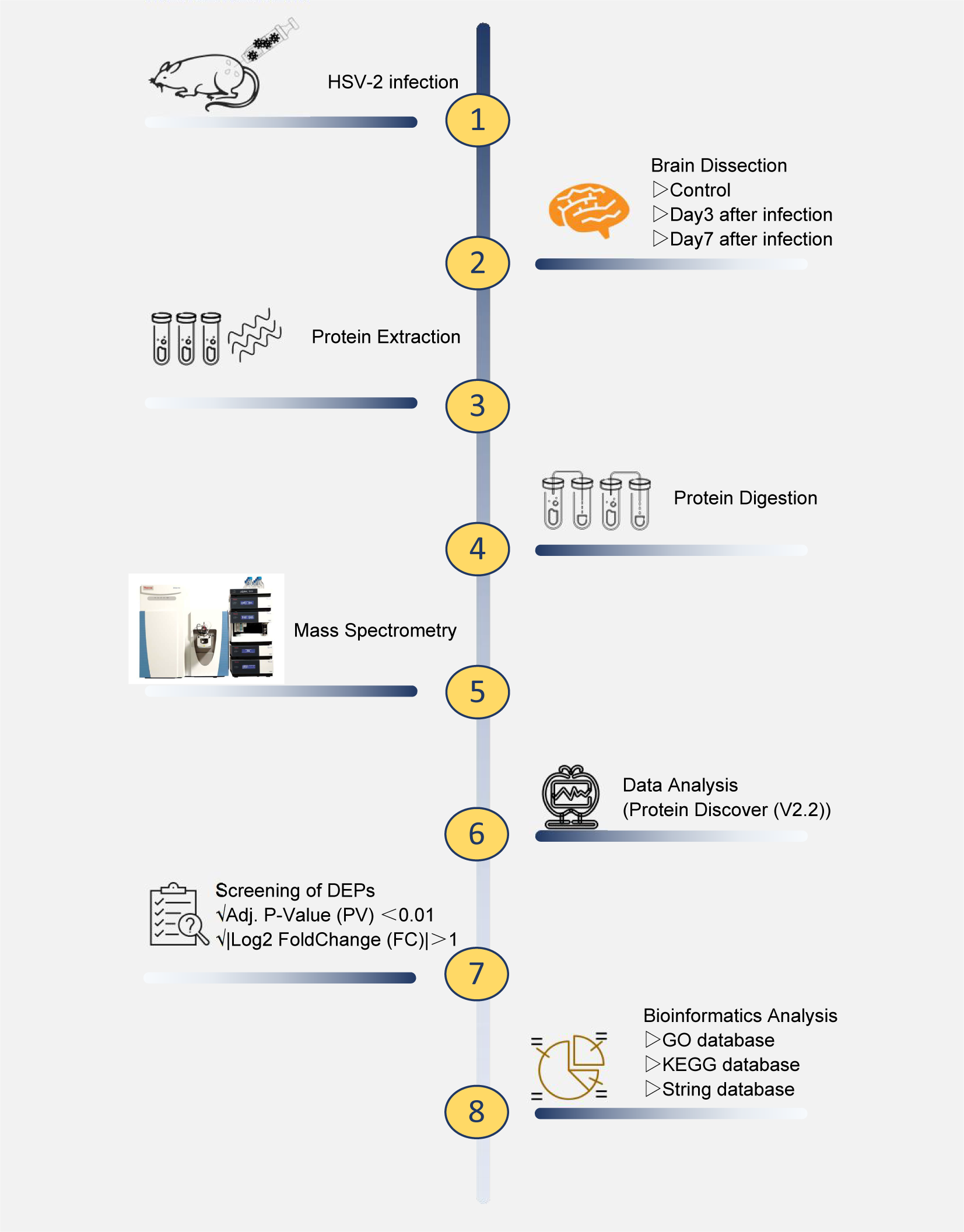

Supplement: Supplementary Figure 3 — Proteomics workflow for label-free quantitation of proteins from brains after vaginal infection with HSV-2. [file Image_3.tif]
