# Supplementary material for: Understanding global changes of the mouse brain proteome after vaginal infection with HSV-2 using a label-free shotgun approach
Source: Front Cell Infect Microbiol. 2022 Aug 18;12:942334. doi: 10.3389/fcimb.2022.942334 (PMC9433710; doi:10.3389/fcimb.2022.942334)
Supplement: Supplementary file 4 [file Table_1.docx]

**Supplement table 1. Proteins differently expressed in brain induced by vaginally infection with HVS-2.**

| Accession | Protein FDR Confidence: Combined | Description | Log2 (MaxLFQ) Day3 / Control | Adj. P-Value Day3 / Control | Log2 (MaxLFQ) Day7 / Control | Adj. P-Value Day7 / Control | MW [kDa] |
| --- | --- | --- | --- | --- | --- | --- | --- |
| **up** |  |  |  |  |  |  |  |
| P12246 | Medium | Serum amyloid P-component | 6.64 | 3.479E-16 | 6.64 | 3.176E-16 | 26.2 |
| Q91X72 | High | Hemopexin | 0.52 | 0.574 | 4.04 | 3.176E-16 | 51.3 |
| A4UUI3 | High | Guanylate binding protein 4.1 | 6.64 | 3.479E-16 | 6.64 | 3.176E-16 | 72 |
| P01902 | High | H-2 class I histocompatibility antigen, K-D alpha chain | 6.64 | 3.479E-16 | 6.64 | 3.176E-16 | 41.5 |
| Q61646 | High | Haptoglobin | 0.20 | 0.942 | 3.21 | 3.176E-16 | 38.7 |
| Q60766 | High | Immunity-related GTPase family M protein 1 | 0.37 | 0.940 | 2.81 | 3.176E-16 | 46.5 |
| A6X935 | High | Inter alpha-trypsin inhibitor, heavy chain 4 | 1.25 | 0.015 | 2.87 | 3.176E-16 | 104.6 |
| P01027 | High | Complement C3 | 0.07 | 0.988 | 2.04 | 3.176E-16 | 186.4 |
| P01901 | High | H-2 class I histocompatibility antigen, K-B alpha chain | 0.02 | 0.969 | 1.71 | 1.517E-11 | 41.3 |
| A0A1B0GT68 | Low | Predicted gene 45717 | 0.18 | 0.988 | 1.61 | 1.019E-08 | 16.7 |
| G3X8T9 | High | Serine (Or cysteine) peptidase inhibitor, clade A, member 3N, isoform CRA_a | 0.17 | 0.988 | 1.37 | 6.455E-08 | 46.7 |
| A0A0R4J038 | High | Kininogen-1 | 0.37 | 0.841 | 1.30 | 5.342E-07 | 73.1 |
| P07759 | High | Serine protease inhibitor A3K | 0.16 | 0.965 | 1.18 | 3.176E-16 | 46.9 |
| P08122 | High | Collagen alpha-2 chain | 0.38 | 0.821 | 1.64 | 5.935E-12 | 167.2 |
| P35436 | High | Glutamate receptor ionotropic, NMDA 2A | 6.64 | 3.479E-16 | 6.64 | 3.176E-16 | 165.3 |
| Q6NSW3 | Medium | A-kinase anchor protein SPHKAP | 6.64 | 3.479E-16 | 6.64 | 3.176E-16 | 185 |
| Q91V36 | High | Nuclear receptor-binding protein 2 | 6.64 | 3.479E-16 | 6.64 | 3.176E-16 | 57.3 |
| P02463 | High | Collagen alpha-1 chain | 0.11 | 1.000 | 1.40 | 1.202E-10 | 160.6 |
| Q99M87 | High | DnaJ homolog subfamily A member 3, mitochondrial | 6.64 | 3.479E-16 | 6.64 | 3.176E-16 | 52.4 |
| Q9ERB0 | High | Synaptosomal-associated protein 29 | 6.64 | 3.479E-16 | 6.64 | 3.176E-16 | 29.6 |
| Q8R123 | High | FAD synthase | 6.64 | 3.479E-16 | 6.64 | 3.176E-16 | 54.7 |
| Q9QUG9 | High | RAS guanyl-releasing protein 2 | 0.70 | 0.486 | 1.42 | 3.344E-05 | 69.4 |
| Q9CZN4 | High | Protein shisa-9 | 0.94 | 0.206 | 1.31 | 0.000 | 46.8 |
| Q01149 | High | Collagen alpha-2(I) chain | 6.64 | 3.479E-16 | 6.64 | 3.176E-16 | 129.5 |
| G3X9Y5 | High | Ubiquitin conjugation factor E4 A | 6.64 | 3.479E-16 | 6.64 | 3.176E-16 | 124.4 |
| Q9CPV9 | Medium | P2Y purinoceptor 12 | 6.64 | 3.479E-16 | 6.64 | 3.176E-16 | 39.4 |
| Q8C547 | High | HEAT repeat-containing protein 5B | 6.64 | 3.479E-16 | 6.64 | 3.176E-16 | 224.2 |
| P36552 | High | Oxygen-dependent coproporphyrinogen-III oxidase, mitochondrial | 1.55 | 0.003 | 1.76 | 2.474E-06 | 49.7 |
| Q8BFT9 | High | Synaptic vesicle 2-related protein | 6.64 | 3.479E-16 | 6.64 | 3.176E-16 | 60.7 |
| Q63918 | High | Caveolae-associated protein 2 | 6.64 | 3.479E-16 | 6.64 | 3.176E-16 | 46.7 |
| F6ZGR6 | High | RIKEN cDNA D430041D05 gene (Fragment) | 6.64 | 3.479E-16 | 6.64 | 3.176E-16 | 181 |
| Q8CC88 | Low | von Willebrand factor A domain-containing protein 8 | 6.64 | 3.479E-16 | 6.64 | 3.176E-16 | 213.3 |
| Q80X85 | High | 28S ribosomal protein S7, mitochondrial | 6.64 | 3.479E-16 | 6.64 | 3.176E-16 | 28 |
| Q91VC9 | High | Growth hormone-inducible transmembrane protein | 6.64 | 3.479E-16 | 6.64 | 3.176E-16 | 37.3 |
| Q99ME2 | Low | WD repeat-containing protein 6 | 6.64 | 3.479E-16 | 6.64 | 3.176E-16 | 121.8 |
| P97411 | Medium | Islet cell autoantigen 1 | 6.64 | 3.479E-16 | 6.64 | 3.176E-16 | 54.3 |
| Q9D0G0 | High | 28S ribosomal protein S30, mitochondrial | 6.64 | 3.479E-16 | 6.64 | 3.176E-16 | 49.9 |
| **down** |  |  |  |  |  |  |  |
| D3Z5G7 | High | Carboxylic ester hydrolase | -1.37 | 2.055E-06 | -6.64 | 3.176E-16 | 62.2 |
| Q8CGA3 | High | Large neutral amino acids transporter small subunit 4 | -1.23 | 0.002 | -6.64 | 3.176E-16 | 62.4 |
| Q8R349 | High | Cell division cycle protein 16 homolog | -0.58 | 0.294 | -6.64 | 3.176E-16 | 71.4 |
| A6H5Y3 | Medium | Methionine synthase | -0.31 | 0.755 | -6.64 | 3.176E-16 | 139 |
| Q61024 | High | Asparagine synthetase [glutamine-hydrolyzing] | -0.25 | 0.787 | -6.64 | 3.176E-16 | 64.2 |
| P0C7L0 | High | WAS/WASL-interacting protein family member 3 | -0.25 | 0.739 | -6.64 | 3.176E-16 | 49.4 |
| Q921S7 | High | 39S ribosomal protein L37, mitochondrial | -0.17 | 0.839 | -6.64 | 3.176E-16 | 48.3 |
| E9Q4S7 | High | Receptor-type tyrosine-protein phosphatase eta | -0.16 | 0.874 | -6.64 | 3.176E-16 | 148.5 |
| Q9CQT7 | High | Desumoylating isopeptidase 1 | -0.09 | 0.909 | -6.64 | 3.176E-16 | 18.4 |
| Q08509 | High | Epidermal growth factor receptor kinase substrate 8 | -0.08 | 0.925 | -6.64 | 3.176E-16 | 91.7 |
| A0A0R4J0T0 | High | Iron-sulfur cluster co-chaperone protein HscB, mitochondrial | -0.07 | 0.934 | -6.64 | 3.176E-16 | 26.7 |
| Q8BS40 | Medium | Ceramide-1-phosphate transfer protein | -0.03 | 0.942 | -6.64 | 3.176E-16 | 24.6 |
| E9QPE7 | High | Myosin-11 | -0.45 | 0.338 | -1.11 | 8.243E-05 | 223.2 |
| Q9ERQ8 | High | Carbonic anhydrase 7 | -1.34 | 0.001 | -2.22 | 5.921E-11 | 29.9 |
| E9Q652 | Medium | Regulator of G-protein-signaling 12 | -0.26 | 0.776 | -1.35 | 0.000 | 156.4 |
| Q64337 | High | Sequestosome-1 | -0.20 | 0.815 | -1.32 | 1.546E-05 | 48.1 |
| Q810U3 | High | Neurofascin | -0.03 | 0.949 | -1.35 | 3.765E-09 | 137.9 |
| P20065 | Medium | Thymosin beta-4 | -1.23 | 0.018 | -2.75 | 9.644E-14 | 5.7 |
| P09925 | High | Surfeit locus protein 1 | -0.09 | 0.914 | -1.29 | 1.191E-08 | 34.8 |
| J3QN31 | High | Adenylosuccinate synthetase isozyme 1 | -0.09 | 0.899 | -2.24 | 3.176E-16 | 52.7 |
| **others** |  |  |  |  |  |  |  |
| Q8VBY2 | Medium | Calcium/calmodulin-dependent protein kinase kinase 1 | -6.64 | 3.479E-16 | -1.76 | 1.425E-07 | 55.8 |
| A0A0B4J1F7 | High | Immunoglobulin-binding protein 1b | -6.64 | 3.479E-16 | -1.15 | 0.002 | 39.2 |
| Q80XK6 | High | Autophagy-related protein 2 homolog B | -6.64 | 3.479E-16 | -0.86 | 0.038 | 231.3 |
| Q78IK4 | High | MICOS complex subunit Mic27 | -6.64 | 3.479E-16 | -0.28 | 0.389 | 29.2 |
| Q8CB44 | High | GRAM domain-containing protein 4 | -6.64 | 3.479E-16 | -0.19 | 0.735 | 72.2 |
| E0CYQ0 | High | MCG13350, isoform CRA_b | -6.64 | 3.479E-16 | -0.01 | 0.958 | 33.3 |
| Q99J77 | High | Sialic acid synthase | -6.64 | 3.479E-16 | -0.01 | 0.923 | 40 |
| P0C7M9 | High | C-type lectin domain family 2 member L | -6.64 | 3.479E-16 | 0.04 | 0.979 | 23.6 |
| Q99K30 | High | Epidermal growth factor receptor kinase substrate 8-like protein 2 | -6.64 | 3.479E-16 | 0.06 | 0.986 | 82.2 |
| Q9DAI2 | High | Intraflagellar transport protein 22 homolog | -6.64 | 3.479E-16 | 0.20 | 0.986 | 20.8 |
| O35544 | High | Excitatory amino acid transporter 4 | -6.64 | 3.479E-16 | 0.24 | 0.909 | 60.7 |
| Q3TE40 | High | Replication protein A 32 kDa subunit | -6.64 | 3.479E-16 | 0.25 | 0.952 | 29.4 |
| B8QI36 | High | Liprin-alpha 4 | -6.64 | 3.479E-16 | 0.30 | 0.938 | 133.6 |
| Q91VM3 | Medium | WD repeat domain phosphoinositide-interacting protein 4 | -6.64 | 3.479E-16 | 0.30 | 0.903 | 39.8 |
| Q6PHQ8 | High | N-alpha-acetyltransferase 35, NatC auxiliary subunit | -6.64 | 3.479E-16 | 0.55 | 0.401 | 83.3 |
| Q9CY57 | High | Chromatin target of PRMT1 protein | -6.64 | 3.479E-16 | 0.67 | 0.112 | 26.6 |
| E9Q8I0 | High | Complement factor H | -6.64 | 3.479E-16 | 4.66 | 3.176E-16 | 141.2 |
| P05366 | High | Serum amyloid A-1 protein | -6.64 | 3.479E-16 | 5.48 | 3.176E-16 | 13.8 |
| E9PXQ7 | High | Protocadherin 10 | -- | -- | 6.64 | 3.176E-16 | 114.9 |
| E9QK04 | High | Neogenin | -- | -- | 6.64 | 3.176E-16 | 162.8 |
| G3X920 | High | Armadillo repeat containing 8, isoform CRA_b | -- | -- | 6.64 | 3.176E-16 | 75.4 |
| P05367 | High | Serum amyloid A-2 protein | -- | -- | 6.64 | 3.176E-16 | 13.6 |
| P62069 | High | Ubiquitin carboxyl-terminal hydrolase 46 | -- | -- | 6.64 | 3.176E-16 | 42.4 |
| P97792 | High | Coxsackievirus and adenovirus receptor homolog | -- | -- | 6.64 | 3.176E-16 | 39.9 |
| Q5NCF2 | Medium | Trafficking protein particle complex subunit 1 | -- | -- | 6.64 | 3.176E-16 | 16.9 |
| Q8R574 | High | Phosphoribosyl pyrophosphate synthase-associated protein 2 | -- | -- | 6.64 | 3.176E-16 | 40.9 |
| Q9D1L9 | High | Ragulator complex protein LAMTOR5 | -- | -- | 6.64 | 3.176E-16 | 9.6 |
| Q64518 | High | Sarcoplasmic/endoplasmic reticulum calcium ATPase 3 | -2.88 | 3.479E-16 | 0.47 | 0.234 | 113.6 |
| E9PV24 | High | Fibrinogen alpha chain | -0.36 | 0.440 | 3.21 | 3.176E-16 | 87.4 |
| O89023 | High | Tripeptidyl-peptidase 1 | -3.22 | 3.479E-16 | 0.12 | 0.994 | 61.3 |
| Q3UH53 | Medium | Protein sidekick-1 | -3.86 | 3.479E-16 | -0.59 | 0.026 | 240.2 |
| Q8CJ40 | High | Rootletin | -0.84 | 0.093 | 2.09 | 5.158E-11 | 226.8 |
| Q8K0E8 | High | Fibrinogen beta chain | -0.07 | 0.917 | 2.72 | 3.176E-16 | 54.7 |
| A0A0N4SWI0 | High | Inositol 1,4,5-trisphosphate receptor type 1 (Fragment) | -2.72 | 3.479E-16 | 0.22 | 0.926 | 24 |
| Q3UER8 | High | Fibrinogen gamma chain | -0.18 | 0.661 | 2.31 | 3.176E-16 | 50.3 |
| Q8CI03 | Low | FLYWCH-type zinc finger-containing protein 1 | -1.98 | 1.897E-06 | 0.39 | 0.876 | 77 |
| F7DBB3 | High | AHNAK nucleoprotein 2 (Fragment) | -0.67 | 0.170 | 1.82 | 6.400E-11 | 166.4 |
| Q61625 | High | Glutamate receptor ionotropic, delta-2 | -2.08 | 3.479E-16 | 0.19 | 0.909 | 113 |
| Q8BXT1 | High | Regulator of G-protein signaling 8 | -2.51 | 3.479E-16 | -0.08 | 0.888 | 21 |
| F7A0B0 | High | Myelin basic protein (Fragment) | -0.22 | 0.747 | 2.24 | 3.176E-16 | 21.3 |
| Q61327 | High | Sodium-dependent dopamine transporter | -1.42 | 1.538E-05 | 0.78 | 0.067 | 68.8 |
| E9PZD8 | High | Ceruloplasmin | -0.50 | 0.206 | 1.47 | 7.197E-09 | 124.1 |
| P03975 | High | IgE-binding protein | -1.56 | 3.507E-08 | 0.39 | 0.667 | 62.7 |
| A0A0R4J081 | High | Complex I intermediate-associated protein 30, mitochondrial | -1.33 | 4.217E-07 | 0.41 | 0.620 | 38 |
| Q9DCR2 | High | AP-3 complex subunit sigma-1 | -1.80 | 2.932E-11 | -0.12 | 0.808 | 21.7 |
| Q02105 | High | Complement C1q subcomponent subunit C | -0.26 | 0.774 | 1.60 | 3.008E-06 | 26 |
| P30681 | High | High mobility group protein B2 | -0.72 | 0.019 | 1.19 | 7.902E-09 | 24.1 |
| E9QP56 | Medium | Apolipoprotein C-III | -1.04 | 0.007 | 0.53 | 0.476 | 15.2 |
| P43275 | High | Histone H1.1 | -1.13 | 6.835E-07 | 0.74 | 0.002 | 21.8 |
| P51910 | High | Apolipoprotein D | -0.28 | 0.673 | 1.47 | 3.406E-09 | 21.5 |
| Q9R171 | High | Cerebellin-1 | -1.16 | 7.963E-07 | 0.33 | 0.620 | 21.1 |
| P43276 | High | Histone H1.5 | -1.28 | 1.255E-09 | 0.17 | 0.736 | 22.6 |
| P11881 | High | Inositol 1,4,5-trisphosphate receptor type 1 | -1.75 | 3.479E-16 | 0.10 | 0.903 | 313 |
| E9QNR6 | Medium | Expressed sequence AI837181 | -1.23 | 3.935E-06 | 0.55 | 0.144 | 31.8 |
| Q9JHW2 | High | Omega-amidase NIT2 | -1.26 | 3.088E-07 | 0.30 | 0.768 | 30.5 |
| Q3V384 | High | AFG1-like ATPase | -0.13 | 0.887 | 1.38 | 8.964E-06 | 54.3 |
| P28651 | High | Carbonic anhydrase-related protein | -1.67 | 3.479E-16 | 0.25 | 0.544 | 33.1 |
| O70551 | Medium | SRSF protein kinase 1 | -1.05 | 0.003 | 0.35 | 0.818 | 73 |
| P04919 | High | Band 3 anion transport protein | -1.13 | 2.026E-07 | 0.38 | 0.337 | 103.1 |
| Q99JP6 | High | Homer protein homolog 3 | -1.64 | 3.479E-16 | 0.09 | 0.967 | 39.7 |
| Q8BW86 | High | Rho guanine nucleotide exchange factor 33 | -1.75 | 8.075E-12 | 0.09 | 0.999 | 94.7 |
| A0A087WSP5 | High | Signal transducer and activator of transcription | -0.17 | 0.813 | 1.23 | 1.262E-05 | 88 |
| Q64471 | High | Glutathione S-transferase theta-1 | -1.47 | 8.310E-05 | -0.12 | 0.869 | 27.4 |
| P14106 | High | Complement C1q subcomponent subunit B | -0.25 | 0.688 | 1.06 | 0.001 | 26.7 |
| Q3V2R3 | High | Beta-chimaerin | -1.48 | 6.425E-08 | 0.20 | 0.958 | 53.8 |
| Q8K4P0 | High | pre-mRNA 3' end processing protein WDR33 | -1.49 | 0.003 | -0.05 | 0.952 | 145.2 |
| P02089 | High | Hemoglobin subunit beta-2 | -1.01 | 3.769E-06 | 0.19 | 0.628 | 15.9 |
| Q61704 | High | Inter-alpha-trypsin inhibitor heavy chain H3 | -0.22 | 0.735 | 1.20 | 1.945E-08 | 99.3 |
| Q3UMR5 | High | Calcium uniporter protein, mitochondrial | -1.23 | 8.937E-07 | -0.20 | 0.617 | 39.7 |
| Q6PA06 | High | Atlastin-2 | -1.07 | 0.004 | -0.03 | 0.925 | 66.2 |
| Q64524 | High | Histone H2B type 2-E | -1.37 | 4.535E-14 | -0.13 | 0.710 | 14 |
| P43277 | High | Histone H1.3 | -1.03 | 2.816E-06 | 0.14 | 0.818 | 22.1 |
| P43274 | High | Histone H1.4 | -1.27 | 2.165E-09 | -0.29 | 0.195 | 22 |
| C0HKE4 | High | Histone H2A type 1-E | -1.76 | 8.785E-11 | -1.05 | 1.091E-06 | 14.1 |
| A2A841 | High | Protein 4.1 | -1.15 | 1.535E-05 | 0.11 | 1.000 | 97.2 |
| Q64522 | High | Histone H2A type 2-B | -1.69 | 1.958E-13 | -0.63 | 0.001 | 14 |
| P02802 | High | Metallothionein-1 | -1.04 | 0.000 | -0.08 | 0.857 | 6 |
| Q3U4N2 | High | Protein-S-isoprenylcysteine O-methyltransferase | -1.25 | 2.055E-06 | 0.20 | 0.967 | 31.9 |
| P10922 | High | Histone H1.0 | -1.24 | 5.292E-09 | -0.34 | 0.087 | 20.8 |
| P12658 | High | Calbindin | -1.12 | 2.205E-07 | -0.26 | 0.312 | 30 |
| Q0VEJ0 | High | Centrosomal protein of 76 kDa | -1.10 | 0.000 | -0.60 | 0.031 | 74.3 |
| Q64669 | Medium | NAD(P)H dehydrogenase [quinone] 1 | -1.18 | 6.078E-06 | -0.70 | 0.007 | 30.9 |
| A0A087WSB8 | High | High mobility group nucleosome-binding domain-containing protein 3 | -1.42 | 0.000 | -1.81 | 3.187E-08 | 13.8 |
| Q80W54 | High | CAAX prenyl protease 1 homolog | -1.16 | 4.695E-05 | -0.92 | 0.001 | 54.7 |
| E9Q7G0 | High | Nuclear mitotic apparatus protein 1 | 1.81 | 9.212E-09 | -- | -- | 235.5 |
| Q8C166 | High | Copine-1 | -0.08 | 0.869 | -1.36 | 4.963E-13 | 58.8 |
| B2RXS4 | Medium | Plexin-B2 | -1.42 | 0.000 | -1.38 | 1.507E-05 | 206.1 |
| Q9QZS3 | High | Protein numb homolog | 6.64 | 3.479E-16 | 6.64 | 3.176E-16 | 70.8 |
| Q8BTX9 | High | Inactive hydroxysteroid dehydrogenase-like protein 1 | 6.64 | 3.479E-16 | 6.64 | 3.176E-16 | 36.8 |
| Q8BPU7 | High | Engulfment and cell motility protein 1 | 0.40 | 0.787 | -1.51 | 9.058E-11 | 83.9 |
| E9Q0H6 | High | Fatty acid-binding protein, brain | 1.12 | 2.040E-06 | 1.02 | 2.120E-12 | 20.6 |
| P47199 | High | Quinone oxidoreductase | 0.27 | 0.928 | -1.00 | 2.385E-05 | 35.2 |
| Q62421 | High | Endophilin-A3 | 1.50 | 0.000 | 0.95 | 0.036 | 38.9 |
| O35449 | High | Proline-rich transmembrane protein 1 | 1.10 | 0.004 | 0.94 | 0.010 | 31.4 |
| Q9CWF2 | High | Tubulin beta-2B chain | 1.07 | 8.032E-06 | 0.86 | 2.975E-09 | 49.9 |
| Q9D0J8 | High | Parathymosin | 0.02 | 0.988 | -1.85 | 3.176E-16 | 11.4 |
| Q8BWS5 | High | G protein-regulated inducer of neurite outgrowth 3 | 1.54 | 0.008 | 1.12 | 0.043 | 80.4 |
| Q8BGS2 | High | BolA-like protein 2 | 1.04 | 0.001 | 0.38 | 0.578 | 10.2 |
| A0A1B0GSJ1 | Medium | TELO2-interacting protein 2 | 6.64 | 3.479E-16 | 6.64 | 3.176E-16 | 59.7 |
| Q8BX70 | High | Vacuolar protein sorting-associated protein 13C | 6.64 | 3.479E-16 | 6.64 | 3.176E-16 | 419.8 |
| Q05186 | High | Reticulocalbin-1 | 1.39 | 0.002 | 0.43 | 0.794 | 38.1 |
| A0A1L1SUI3 | High | Solute carrier family 37 (glucose-6-phosphate transporter), member 4 | 6.64 | 3.479E-16 | 6.64 | 3.176E-16 | 48.4 |
| Q810J8 | High | Zinc finger FYVE domain-containing protein 1 | 2.06 | 8.929E-05 | 1.90 | 2.482E-06 | 86.9 |
| Q64018 | Medium | Glycine receptor subunit alpha-1 | 6.64 | 3.479E-16 | 6.64 | 3.176E-16 | 52.6 |
| Q9D164 | High | FXYD domain-containing ion transport regulator 6 | 6.64 | 3.479E-16 | 6.64 | 3.176E-16 | 10.4 |
| A2A7F6 | High | Chloride channel protein | 1.56 | 0.002 | 0.59 | 0.609 | 97.1 |
| P28667 | High | MARCKS-related protein | 1.21 | 9.233E-09 | 0.69 | 1.674E-05 | 20.2 |
| Q8VD75 | High | Huntingtin-interacting protein 1 | 6.64 | 3.479E-16 | 6.64 | 3.176E-16 | 115.1 |
| E9PWE8 | High | Dihydropyrimidinase-related protein 3 | 1.39 | 5.256E-10 | 0.83 | 1.191E-08 | 73.8 |
| Q9DAM7 | Medium | Transmembrane protein 263 | 6.64 | 3.479E-16 | 6.64 | 3.176E-16 | 11.5 |
| O35417 | Medium | Proenkephalin-B | 1.76 | 0.000 | 1.50 | 0.001 | 28 |
| Q9D2R0 | High | Acetoacetyl-CoA synthetase | 1.60 | 1.768E-06 | 1.05 | 0.003 | 75.2 |
| Q91VA6 | High | Polymerase delta-interacting protein 2 | 6.64 | 3.479E-16 | 6.64 | 3.176E-16 | 41.8 |
| P62960 | High | Nuclease-sensitive element-binding protein 1 | 1.24 | 0.000 | 0.70 | 0.132 | 35.7 |
| P70245 | High | 3-beta-hydroxysteroid-Delta(8),Delta(7)-isomerase | 1.29 | 0.000 | 0.34 | 0.825 | 26.2 |
| A0A140LIT2 | High | 7-dehydrocholesterol reductase | 2.42 | 1.786E-11 | 1.52 | 2.021E-05 | 54.2 |
| Q6ZPQ6 | High | Membrane-associated phosphatidylinositol transfer protein 2 | 0.29 | 0.928 | -1.22 | 3.989E-06 | 147.9 |
| E9PUL5 | High | Proline-rich transmembrane protein 2 | 0.26 | 0.842 | -1.34 | 3.176E-16 | 35.9 |
| Q7TNV0 | High | Protein DEK | 1.77 | 6.056E-08 | 0.93 | 0.007 | 43.1 |
| Q64676 | High | 2-hydroxyacylsphingosine 1-beta-galactosyltransferase | 2.11 | 6.391E-11 | 1.53 | 1.438E-07 | 61.2 |
| P14602 | Medium | Heat shock protein beta-1 | 0.13 | 0.988 | -1.13 | 0.004 | 23 |
| E9PW66 | High | Nucleosome assembly protein 1-like 1 | 0.41 | 0.786 | -1.04 | 4.598E-06 | 48.5 |
| Q8JZK9 | High | Hydroxymethylglutaryl-CoA synthase, cytoplasmic | 1.21 | 2.586E-05 | 0.05 | 0.982 | 57.5 |
| Q32NY4 | High | Metal transporter CNNM3 | 0.17 | 1.000 | -1.06 | 0.007 | 76.2 |
| Q3UV17 | High | Keratin, type II cytoskeletal 2 oral | 1.68 | 2.476E-13 | -0.04 | 0.903 | 62.8 |
| Q6A0D4 | High | Raftlin | 0.21 | 0.988 | -1.15 | 0.001 | 61.5 |
| Q80X90 | High | Filamin-B | 0.42 | 0.602 | -1.14 | 1.989E-08 | 277.7 |
| O70456 | High | 14-3-3 protein sigma | 1.90 | 1.379E-05 | 0.46 | 0.814 | 27.7 |
| Q8K2C7 | High | Protein OS-9 | 0.08 | 0.983 | -1.25 | 0.001 | 76.1 |
| P28184 | High | Metallothionein-3 | 0.76 | 0.034 | -1.38 | 4.950E-12 | 7 |
| A0A0U1RP81 | High | MICOS complex subunit MIC60 | 0.40 | 0.787 | -1.38 | 2.511E-08 | 53.7 |
| Q5XKN4 | High | Protein jagunal homolog 1 | 0.24 | 0.956 | -1.57 | 1.480E-08 | 21.1 |
| Q8BGV8 | High | Mitochondrial dynamics protein MID51 | 0.70 | 0.653 | -1.21 | 0.001 | 51.2 |
| Q9D2R8 | Medium | 28S ribosomal protein S33, mitochondrial | 0.71 | 0.657 | -1.21 | 0.002 | 12.5 |
| D3Z4S3 | High | Putative peptidyl-tRNA hydrolase PTRHD1 | 0.53 | 0.680 | -1.40 | 1.347E-06 | 16 |
| Q8BJU2 | High | Tetraspanin-9 | 0.64 | 0.754 | -1.41 | 0.000 | 26.7 |
| E9Q557 | High | Desmoplakin | 1.39 | 0.008 | -0.26 | 0.667 | 332.7 |
| Q922H4 | High | Mannose-1-phosphate guanyltransferase alpha | 0.28 | 0.928 | -1.80 | 3.673E-11 | 46.2 |
| P70182 | Medium | Phosphatidylinositol 4-phosphate 5-kinase type-1 alpha | 0.03 | 0.969 | -2.06 | 2.532E-12 | 60.4 |
| Q9CPX6 | Medium | Ubiquitin-like-conjugating enzyme ATG3 | 0.30 | 0.958 | -1.78 | 5.356E-09 | 35.8 |
| Q9QZB7 | High | Actin-related protein 10 | 0.34 | 0.855 | -1.92 | 3.176E-16 | 46.2 |
| A8DUK4 | High | Beta-globin | 6.64 | 3.479E-16 | 6.64 | 3.176E-16 | 15.7 |
| E9Q1U6 | High | CBP80/20-dependent translation initiation factor | 0.15 | 0.980 | -2.34 | 3.176E-16 | 70.2 |
| P60761 | High | Neurogranin | 2.88 | 3.479E-16 | -0.49 | 0.043 | 7.5 |
| G3X8R8 | Low | MCG123888 | 1.31 | 0.087 | -1.89 | 8.570E-07 | 118.5 |
| Q8VCG1 | High | Deoxyuridine triphosphatase | 1.37 | 0.003 | -2.43 | 3.216E-13 | 21.2 |
| Q8VHL0 | High | Urea transporter 1 | 0.01 | 0.965 | -3.90 | 3.176E-16 | 42.1 |
| P56475 | Low | Gamma-aminobutyric acid receptor subunit rho-1 | 0.56 | 0.691 | -3.88 | 3.176E-16 | 55.5 |
| Q9DCZ4 | High | MICOS complex subunit Mic26 | 0.05 | 0.983 | -4.41 | 3.176E-16 | 22.6 |
| Q99LD9 | High | Translation initiation factor eIF-2B subunit beta | 2.89 | 4.812E-11 | -2.24 | 2.313E-09 | 38.9 |
| J3QNY1 | High | Predicted pseudogene 9242 | 0.20 | 0.949 | -2.27 | 3.176E-16 | 37.1 |
| Q9D0L7 | Medium | Armadillo repeat-containing protein 10 | 1.35 | 0.011 | -4.61 | 3.176E-16 | 33.3 |
| Q8BFY6 | High | Peflin | 0.05 | 0.986 | -6.64 | 3.176E-16 | 29.2 |
| P00158 | High | Cytochrome b | 0.21 | 0.942 | -6.64 | 3.176E-16 | 43.2 |
| Q9Z1K5 | High | E3 ubiquitin-protein ligase ARIH1 | 0.36 | 0.837 | -6.64 | 3.176E-16 | 64 |
| Q8CDM8 | Medium | Protein FAM160B1 | 0.38 | 0.809 | -6.64 | 3.176E-16 | 86 |
| O35075 | High | Down syndrome critical region protein 3 homolog | 0.43 | 0.883 | -6.64 | 3.176E-16 | 32.9 |
| P15532 | High | Nucleoside diphosphate kinase A | 0.51 | 0.255 | -6.64 | 3.176E-16 | 17.2 |
| Q9D074 | High | E3 ubiquitin-protein ligase MGRN1 | 0.53 | 0.870 | -6.64 | 3.176E-16 | 58.4 |
| Q8BI72 | High | CDKN2A-interacting protein | 0.57 | 0.837 | -6.64 | 3.176E-16 | 59.7 |
| Q91YP0 | High | L-2-hydroxyglutarate dehydrogenase, mitochondrial | 0.57 | 0.427 | -6.64 | 3.176E-16 | 50.9 |
| A0A140LHG8 | High | Signal peptidase complex subunit 2 | 0.59 | 0.783 | -6.64 | 3.176E-16 | 28.4 |
| Q9CQM5 | High | Thioredoxin domain-containing protein 17 | 0.64 | 0.394 | -6.64 | 3.176E-16 | 14 |
| Q7TSF1 | High | Desmoglein-1-beta | 0.78 | 0.471 | -6.64 | 3.176E-16 | 114.4 |
| Q8R1G2 | High | Carboxymethylenebutenolidase homolog | 0.88 | 0.071 | -6.64 | 3.176E-16 | 27.9 |
| P63168 | High | Dynein light chain 1, cytoplasmic | 1.01 | 0.019 | -6.64 | 3.176E-16 | 10.4 |
| A0A1D5RLT6 | High | GRAM domain-containing protein 1B | 1.14 | 0.102 | -6.64 | 3.176E-16 | 88.7 |
| O08807 | High | Peroxiredoxin-4 | 1.15 | 0.006 | -6.64 | 3.176E-16 | 31 |
| F7C3A0 | High | Phosphoinositide phospholipase C | 1.89 | 0.001 | -6.64 | 3.176E-16 | 136.2 |
| P24529 | High | Tyrosine 3-monooxygenase | 2.05 | 0.000 | -6.64 | 3.176E-16 | 56 |
| Q91XU0 | High | ATPase WRNIP1 | 2.44 | 3.179E-06 | -6.64 | 3.176E-16 | 71.7 |
| P56565 | High | Protein S100-A1 | 3.03 | 3.479E-16 | -6.64 | 3.176E-16 | 10.5 |
| K3W4Q5 | Medium | Family with sequence similarity 186, member A | 4.00 | 3.479E-16 | -6.64 | 3.176E-16 | 331.3 |
| Q91YH5 | High | Atlastin-3 | 5.17 | 3.479E-16 | -6.64 | 3.176E-16 | 60.5 |
| Q62093 | High | Serine/arginine-rich splicing factor 2 | 1.79 | 1.170E-08 | -1.78 | 8.985E-14 | 25.5 |
| A0A0R4J1J1 | High | MCG114807, isoform CRA_c | 6.64 | 3.479E-16 | -- | -- | 15.6 |
| F6TQW2 | Medium | Immunoglobulin heavy constant gamma 2C (Fragment) | 6.64 | 3.479E-16 | -- | -- | 44.2 |
| G3X9N1 | Medium | Rho GTPase activating protein 24, isoform CRA_b | 6.64 | 3.479E-16 | -- | -- | 84.1 |
| Q08189 | High | Protein-glutamine gamma-glutamyltransferase E | 6.64 | 3.479E-16 | -- | -- | 77.3 |
| Q0VGY8 | High | Protein TANC1 | 6.64 | 3.479E-16 | -- | -- | 200.7 |
| Q8BXK8 | High | Arf-GAP with GTPase, ANK repeat and PH domain-containing protein 1 | 6.64 | 3.479E-16 | -- | -- | 94.4 |
| Q8CFI0 | High | E3 ubiquitin-protein ligase NEDD4-like | 6.64 | 3.479E-16 | -- | -- | 115.3 |
| Q9QXG2 | Medium | Rab proteins geranylgeranyltransferase component A 1 | 6.64 | 3.479E-16 | -- | -- | 73.9 |
| A8IK50 | High | Purkinje cell protein 2 | -6.64 | 3.479E-16 | -6.64 | 3.176E-16 | 14.8 |
| P35550 | High | rRNA 2'-O-methyltransferase fibrillarin | -6.64 | 3.479E-16 | -6.64 | 3.176E-16 | 34.3 |
| P61963 | High | DDB1- and CUL4-associated factor 7 | -6.64 | 3.479E-16 | -6.64 | 3.176E-16 | 38.9 |
| Q8BLV3 | High | Sodium/hydrogen exchanger 7 | -6.64 | 3.479E-16 | -6.64 | 3.176E-16 | 80.2 |
| Q91YE6 | High | Importin-9 | -6.64 | 3.479E-16 | -6.64 | 3.176E-16 | 116 |
| Q922B1 | High | O-acetyl-ADP-ribose deacetylase MACROD1 | -6.64 | 3.479E-16 | -6.64 | 3.176E-16 | 35.3 |
| Q99MR0 | Low | Actin-like protein 6B | -6.64 | 3.479E-16 | -6.64 | 3.176E-16 | 46.9 |
| Q9D6E4 | Medium | Tetratricopeptide repeat protein 9B | -6.64 | 3.479E-16 | -6.64 | 3.176E-16 | 25.9 |
| Q9JMG1 | High | Endothelial differentiation-related factor 1 | -6.64 | 3.479E-16 | -6.64 | 3.176E-16 | 16.4 |
